# Soil Seedbank Dynamics and Species Diversity in *Pimelea*-Infested Paddocks Under Pasture and Cultivated Conditions

**DOI:** 10.3390/biology14020109

**Published:** 2025-01-21

**Authors:** Rashid Saleem, Ali Bajwa, Shane Campbell, Mary T. Fletcher, Sundaravelpandian Kalaipandian, Steve W. Adkins

**Affiliations:** 1 School of Agriculture and Food Sustainability, The University of Queensland, Gatton, QLD 4343, Australia; shane.campbell@uq.edu.au (S.C.); s.adkins@uq.edu.au (S.W.A.); 2 La Trobe Institute of Sustainable Agriculture and Food (LISAF), Department of Ecological, Plant and Animal Sciences, AgriBio, La Trobe University, Melbourne, VIC 3086, Australia; a.bajwa@latrobe.edu.au; 3 Queensland Alliance for Agriculture and Food Innovation, The University of Queensland, Coopers Plains, QLD 4108, Australia; mary.fletcher@uq.edu.au; 4 Department of Bioengineering, Saveetha Institute of Medical and Technical Sciences (SIMATS), Saveetha School of Engineering, Chennai 602105, Tamil Nadu, India

**Keywords:** *P. trichostachya*, composition, seedbank, species richness, diversity, pasture, Shannon–Wiener index

## Abstract

Pasture lands, while appearing uniform in species diversity, exhibit notable variations upon closer examination. The study on *Pimelea* (or riceflower) seedbank dynamics revealed significant variations in seed density and distribution across soil depths, sites, years, and between pasture and cultivated paddocks in the same region of western Queensland. For the total number of germinable riceflower seeds across both the years 2019 and 2020, there is a clear distinction between pasture and cultivated paddocks. Pasture paddocks exhibited a gradual increase in total seed count from 108 seeds m^−2^ in 2019 to 121 seeds m^−2^ in 2020, resulting in a combined total of 229 seeds m^−2^ over the 2 years across both depths. In contrast, cultivated paddocks showed a more substantial increase, with seed counts rising from 146 seeds m^−2^ in 2019 to 255 seeds m^−2^ in 2020, resulting in a combined total of 401 seeds m^−2^ across both depths. Additionally, the Shannon–Wiener index at Site 1 indicated increased species diversity in the topsoil of pasture paddocks in 2020 compared to 2019, while deeper soil diversity decreased. Cultivated paddocks showed a declining trend, while pasture sites exhibited stable or increasing diversity. Pasture management generally maintained or enhanced diversity better than cultivation, especially in the topsoil layer. These findings highlight site-specific differences influenced by soil properties, land management practices, and local environmental conditions, shaping riceflower seedbank dynamics. Understanding these patterns is crucial for developing targeted management strategies to control riceflower in affected areas. Long-term field studies focusing on seedbanks are essential to develop sustainable control strategies.

## Simple Summary

This study looked at how pasture and cultivated lands in western Queensland impact riceflower spread and plant diversity. Researchers found that pasture areas tended to support a greater variety of plant species, especially near the soil surface, while cultivated lands had more riceflower seeds, mostly deeper in the soil. This difference suggests that farming practices, which disturb the soil, may encourage riceflower growth but reduce other plant diversity. Seed distribution also changed with climate conditions from year to year, highlighting the need to consider weather patterns in planning land use. By understanding these patterns, farmers and land managers can adopt practices that help control riceflower while preserving soil health and plant diversity. These findings emphasize the importance of sustainable land management practices that balance agricultural productivity with ecosystem preservation. Ultimately, this research provides valuable insights for developing effective land use strategies that benefit both the environment and local communities by supporting resilient, diverse ecosystems.

## 1. Introduction

The soil seedbank serves as a reservoir for future vegetation and reflects the past aboveground plant life in the land area [1]. Viable seeds are distributed across the land surface and throughout its profile [2,3]. Plants reproduce by producing seeds, which can persist in the soil and contribute to the soil seedbank [4]. Weeds invading communities often have a profound effect on the size and makeup of the soil seedbank [5]. *Pimelea*, collectively known as riceflowers, is a genus belonging to the family Thymelaeaceae, and has become a significant agricultural concern due to its toxicity to livestock, particularly cattle (*Bos taurus* L., *B. indicus* L.) [6]. Three noxious species, *Pimelea trichostachya* Lindl., *P. simplex* F. Muell., and *P. elongata* Threlfall, are of particular concern due to their production of the toxin simplexin. This toxin can lead to the death of cattle and severely weaken surviving animals, reducing productivity and pasture carrying capacity [6].

Once *Pimelea* seeds are dispersed from the parent plant, they often become trapped by stones, sticks, earth clods, stumps, or bushes. These natural barriers facilitate propagation under favourable climatic conditions. However, fresh seeds of the three toxic *Pimelea* species (*P. simplex*, *P. trichostachya*, and *P. elongata*) are typically dormant and do not germinate immediately. This dormancy is primarily due to their thick fruit/seed coat, which resists water absorption. For germination to begin, a process of weathering is required to break down these protective layers [7]. Fresh *Pimelea* seeds possess a thin membrane (mesocarp) between the hairy outer seed coat (exocarp) and the inner seed coat (endocarp), which is impermeable to water. Nevertheless, each seed contains a tiny pore that allows limited moisture to penetrate. A study conducted between 2007 and 2009 examined weathering effects on *P. simplex* subsp. *continua*, *P. trichostachya*, and *P. elongata* under field conditions in South Australia, New South Wales, and Queensland. The findings revealed that after several months of exposure to natural environmental conditions, the germination rates of *P. trichostachya* and *P. elongata* improved significantly compared to seeds stored under controlled laboratory conditions. This highlights the importance of environmental weathering in breaking seed dormancy and initiating germination [7]. The ingestion of *Pimelea* tissues causes a condition commonly known as *Pimelea* poisoning which can lead to symptoms such as weight loss, diarrhoea, and even death in severely affected animals [7].

**Figure 1:**
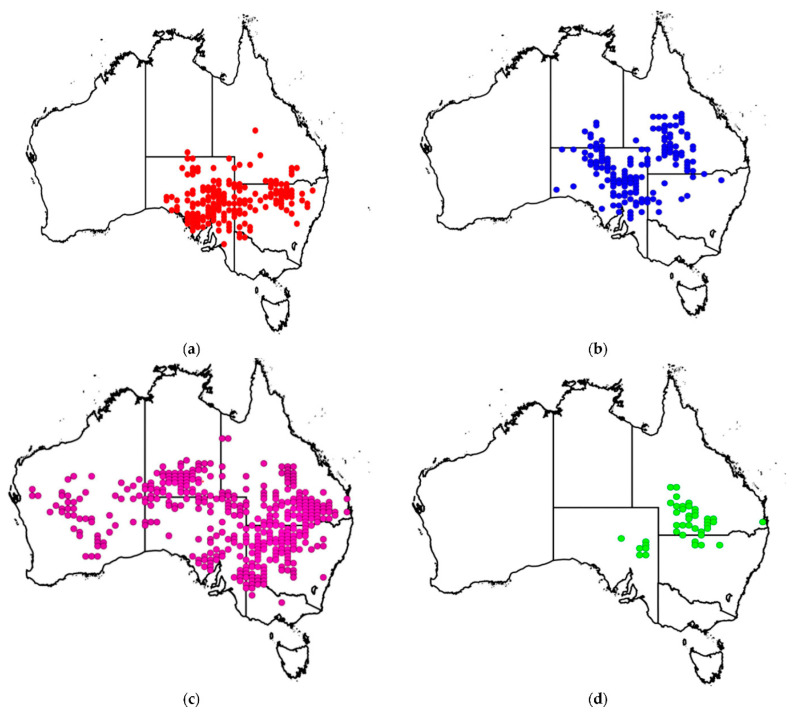
Distribution maps of four poisonous *Pimelea* species across Australian States and Territories: (**a**) *Pimelea simplex* subsp. *simplex*, (**b**) *Pimelea simplex* subsp. *continua*, (**c**) *Pimelea trichostachya*, and (**d**) *Pimelea elongata*.

Losses due to poisoning are by far the most significant and have become a major threat to the viability of the livestock industry in certain areas. The financial impact is substantial to the livestock industry. A recent survey on *Pimelea* infestations (2021) revealed that affected producers reported average annual financial losses of about AUD 67,000. Additionally, 50% of respondents experienced an average of 26 cattle deaths annually. Some landholders also reported spending up to AUD $2100 per year on *Pimelea* control measures, underscoring the substantial economic burden this genus imposes on the agricultural sector [6]. *Pimelea trichostachya* Lindl. is an annual forb that typically grows 20 to 60 cm high, has a bisexual mode of reproduction, and is widespread across all Australia (Figure 1). Previous studies have reported that this species prefers to grow on sandy soils with different acidity and colour and on hard-setting duplex soils. *Pimelea trichostachya* can grow well in dense buffel grass [*Pennisetum ciliare* L. (Link.)] pasture [7].

Agronomic practices, such as cultivation, grazing, and the use of herbicides, can significantly alter seedbank composition and dynamics. For instance, practices like soil disturbance through tillage may bury seeds deeper into the soil, potentially extending their viability while simultaneously delaying germination. Conversely, surface disturbance can stimulate seed germination, depleting the seedbank in the short term but risking rapid weed proliferation if not managed effectively. The timing and frequency of these practices further influence their impact, with poorly timed interventions potentially exacerbating the problem by promoting seed dispersal or recruitment. According to Fletcher [8], cultivation can bury seeds, potentially prolonging their viability. This implies that cultivation and fallowing are unlikely to reduce the potential recruitment of riceflower unless cultivation occurs at the vegetative stage, before flowering. However, frequent cultivation can lead to soil degradation, erosion, and loss of soil structure, negatively impacting soil health and community biodiversity [9] and further reducing pasture productivity [10].

One of the most dominant species of *Pimelea* in the grazing lands of southwestern Queensland is the annual riceflower (*P. trichostachya* Lindl.). A single annual riceflower plant can produce several hundred seeds, which can result in the formation of large seedbanks that remain viable for up to 2 years [11]. The dynamics of riceflower seedbanks are poorly studied until now. Previous studies revealed that land disruption activities like land clearing, cultivation, and disturbance by domestic animals could stimulate the germination of a seedbank [12]. Further, the germination rates from seedbanks improved over the course of time [8], which indicates the presence of dormancy in the freshly shed seeds. In addition, the annual riceflower seeds buried deep in the seedbank appear to extend their viability as they are protected from predatory surface agents [8], but seedling emergence is difficult from depths greater than 3 cm in the soil seedbank [6].

Community diversity is crucial for designing effective weed management strategies, as it considers a weed as part of an ecological community rather than as an isolated species [13]. Assessing this diversity provides valuable insights into the challenges of managing a specific weed within a landscape. Various indices, such as the Shannon–Wiener Index (SWI) [14] which measures biodiversity, account for diversity, richness, abundance, and evenness of species present in a community [15,16]. The SWI is particularly effective for comparative analyses of biodiversity across various environmental conditions, weed management approaches, and soil depths during seedbank examinations. Seedbanks in annual riceflower-infested areas are poorly studied, leaving landholders with a poor understanding of such seedbanks.

A thorough understanding of seedbank dynamics provides insights into the lifecycle of target weeds and their interactions with the surrounding ecosystem. This knowledge enables the design of integrated weed management programs that optimize agronomic practices to reduce weed seedbank replenishment, enhance seedbank depletion, and promote the establishment of competitive, desirable plant species. By addressing both immediate and long-term weed control objectives, such strategies can contribute to sustainable agricultural productivity, improved pasture health, and reduced economic losses for farmers. Properties with varying levels of riceflower infestation were prioritized to capture the full spectrum of its impact on pasture productivity and livestock health. For this study, we visited over eight properties, encompassing a total area of approximately 161,943 ha; however, we focused on two key sites, selected based on the severity of riceflower infestation, accessibility, and farmers’ willingness to collaborate. Temporal and spatial considerations were kept in mind to evaluate changes in infestation patterns across different years and locations. By comparing data across years, we can better understand the dynamics of riceflower infestations and develop more effective mitigation strategies. These findings contribute to improving farm management practices and minimizing economic losses for the broader agricultural sector.

This study aims to fill the gap in knowledge regarding *Pimelea* seedbank dynamics, focusing on their impact on pasture productivity and livestock health. We hypothesize that *Pimelea* maintains a seedbank that responds sensitively to environmental conditions, with a significant proportion of seeds expected to germinate under favourable conditions, resulting in sporadic population surges. By analysing seedbank characteristics and assessing the impact of cultivation, this study seeks to provide a better understanding of how agronomic practices affect the long-term management of annual riceflower. With a focus on two sites in southwestern Queensland, we aim to offer new insights into the seedbank dynamics of *P. trichostachya* and to evaluate the role of cultivation in its management. This research is crucial not only for improving farm management practices but also for minimizing the economic losses caused by *Pimelea* infestations across the broader agricultural sector.

## 2. Materials and Methods

### 2.1. Site Description

The two study sites heavily infested by annual riceflower were located on rural properties in the Maranoa shire of southwestern Queensland (QLD). At each site, adjacent pasture and crop paddocks on similar soil types were selected for seedbank sampling. Site 1 [27°38′6″ S, 148°42′29″ E (pasture paddock); 27°38′21″ S, 148°42′43″ E (cultivated paddock)] was approximately 55 km away from the town of St. George, while Site 2 [27°15′32″ S, 148°34′26″ E (pasture land); 27°13′9″ S, 148°32′58″ E (cultivated land)] was approximately 65 km away from Surat. The distance between the two sites was roughly 35 km.

The pasture paddock at Site 1 had a rich variety of grasses, including buffel grass (*Pennisetum ciliare* L.); the soil had a pH of 6.2, an organic matter content of 2.0%, a carbon content of 1.4%, a nitrogen content of 0.1%, a phosphorus content of 14 mg kg^−^^1^, a sulphur content of 3.7 mg kg^−^^1^, and a potassium content of 443 mg kg^−^^1^ (Table 1). The cultivated paddock also had a similar constitution, apart from having more phosphorus (21.4 mg kg^−^^1^) and sulphur (3.7 mg kg^−^^1^; Table 1).

At Site 2, the pasture paddock soil had buffel grass *(Pennisetum ciliare* L.), and a pH of 6.0, an organic matter content of 1.8%, a carbon content of 1.32%, a nitrogen content of 0.09%, a phosphorus content of 23.2 mg kg^−^^1^, a sulphur content of 2.6 mg kg^−^^1^, and a potassium content of 371 mg kg^−^^1^ (Table 1). The cultivated paddock soil at Site 2 had similar characteristics but with higher organic matter (2.3%), potassium (380 mg kg^−^^1^), phosphorus (43.8 mg kg^−^^1^), nitrogen (0.12%), and carbon (2.0%) compared to the pasture soil. The sulphur content in the cultivated soil remained the same at 2.8 mg kg^−^^1^ (Table 1). Site 1 was fallow for four years, allowing soil recovery through natural processes like nutrient recycling and weed suppression. Site 2, kept fallow for 5 years, had an additional year to achieve improved soil health, enhancing organic matter, soil structure, and microbial activity. While both sites benefited, the extra year at Site 2 likely resulted in greater improvements in soil fertility and structure (farmer communication).

From 2018 to 2020, both study sites experienced typical seasonal changes in rainfall and temperature (Table 2 and Table 3); however, there were some differences between the years. Site 1 had an annual rainfall of 408 mm in 2018, a notably dry year in 2019 with 153 mm, and a return to a typical annual rainfall of 422 mm in 2020. The peaks in rainfall were in the periods February to March and October to November for the three years. Similarly, Site 2 had an annual rainfall of 337 mm in 2018, a notably dry year in 2019 with 152 mm, and a return to a typical to high annual rainfall of 646 mm in 2020, again with peaks in rainfall in the February to March and October to November periods in the 3 years as at Site 1 (Table 2). Lower rainfall and increasing evaporation will cause more frequent depletion of soil moisture, reduced ground cover, and lower livestock carrying capacity (Queensland Government).

Temperature data revealed that Site 1 was marginally warmer than Site 2, with both sites reaching their maximum annual temperatures in January (35.3 °C at Site 1; 34.4 °C at Site 2) and their lowest in July (19.7 °C at Site 1; 19.9 °C at Site 2). Minimum temperatures also followed this pattern, being highest in January and lowest in July (Table 3).

### 2.2. Soil Seedbank Collection and Analysis

During 2019 and 2020, soil seedbank samples were collected in September from both pasture and cultivated paddocks. For each collection, 10 quadrats (1 m × 1 m) were positioned along a 50 m transect line, with the quadrats spaced 5 m apart. From each quadrat, five cylindrical soil cores, each 10 cm in diameter, were extracted using a soil corer; one sample was taken from each corner of the quadrat and one from its centre. Each soil core upon removal was divided into two portions: from soil surface to 5.0 cm deep and from 5.0 to 10.0 cm deep in the pasture paddock, and from soil surface to 7.5 cm deep and from 7.5 to 15.0 cm deep in the cultivated paddock. The soil depth differences between the pasture and cultivated paddocks result from the way each is managed. In the pasture paddock, soil from 0 to 5 cm and 5 to 10 cm is more compact due to grazing animals. In the cultivated paddock, ploughing loosens the soil more deeply, affecting the layers from 0 to 7.5 cm and 7.5 to 15 cm. The five soil samples from each quadrat were then pooled, creating two samples, one for each depth. Then, all upper and all lower soil core samples from the 10 quadrats were pooled separately and placed into sealed and labelled plastic bags, returned to the laboratory, and stored at ambient temperature for 2 to 3 days in preparation for the seedbank germination trial.

Ten soil seedbank samples from two sites, two depths, and two land uses for a total of 80 samples were collected each year. These subsamples were individually spread thinly over a 2 cm layer of sterilized UQ Gatton media (Osmocote 8–9 M, Osmocote 3–4 M, and Nutricote 7 M, containing coated iron, a moisture aid, dolomite, and Osmoform) in shallow trays (25 cm × 20 cm × 6 cm; length × width × depth). The trays were distributed randomly on a series of benches in a polytunnel located at The University of Queensland, Gatton Campus. The trays were watered regularly to maintain optimal moisture conditions for seed germination and growth.

Among the experimental trays containing the soil samples, two control trays were strategically placed to monitor for any contaminating seeds present in the compost or the greenhouse environment (no contaminating seedlings were found during the experiment). The sample trays were observed regularly for newly emerging seedlings. Each seedling upon emergence was marked with a coloured wooden toothpick and initially categorized as either ‘*Pimelea*’ or ‘other’ species. Once the seedlings grew large enough for identification, they were removed from the trays. For those seedlings that were not easily identifiable, representative individuals were transferred to small pots containing potting compost and grown to maturity, then identified. The observations continued until the emergence of seedlings ceased, after about 6 weeks. Subsequently, the soil and compost were allowed to dry for 2 weeks, then thoroughly stirred, and rewetted back to field capacity. Samples were then re-examined over the course of another 4 weeks to check for any further seedling emergence. This procedure of drying and rewetting was repeated until no more seedlings emerged.

### 2.3. Shannon–Weiner Index (SWI)

Diversity, abundance, evenness, and species richness were determined for each of the soil seedbank samples taken from the two sites (viz. cultivated and pasture paddocks) and two soil depths during the 2 years of the study. Diversity was determined as the number of different species present in the sample, while species abundance and evenness were determined by the SWI [14].

$$\mathrm{Shannon}–\mathrm{Weiner}\;\mathrm{index}\;(H)=-\sum_{i=1}^{S}pi.\mathrm{In}\;pi\;$$

$$\mathrm{Shannon}–\mathrm{Weiner}\;\mathrm{index}\;(H)=-\sum_{i=1}^{S}\left(\frac{\mathrm{ni}}{N}.\;\mathrm{In}\frac{\mathrm{ni}}{N}\;\right)$$

where *pi* (ni/N) represents the proportion of individuals of species (i) relative to the total number of individuals present in the community. The index is a measure of the relative abundance of each species where N is the total number of individuals of all species, ln is the natural logarithm, ni is the number of individuals of the i-th species, and S is the total number of species in the community. A comparison of the calculated values for the different situations [13] indicates which of the conditions is most diverse (values of < 1.0 indicate low diversity; values > 1.0 indicate high diversity). If a community is dominated by a certain species, the index will be low.

The number of germinable seeds per m^2^ is determined by dividing the number of viable seeds capable of germinating within a sample area by the size of that area. Sample area indicates the physical boundaries of the sampled soil, typically measured in square meters (m^2^). This is the area from which the soil sample was collected.

$$\mathrm{Number}\;\mathrm{of}\;\mathrm{germinable}\;\mathrm{seeds}=\frac{\mathrm{Number}\;\mathrm{of}\;\mathrm{germinable}\;\mathrm{seeds}\;\mathrm{in}\;\mathrm{soil}\;\mathrm{core}}{\mathrm{Size}\;\mathrm{of}\;\mathrm{area}\;\left(m^{2}\right)}$$

The average population size per m^2^ was calculated by dividing the total number of individuals counted in all sampled plots by the total area of those plots. This measure provides an estimate of the density of individuals across various species within a given habitat. It is widely used in population ecology to assess species distribution, community structure, and population density.

$$\mathrm{Average}\;\mathrm{population}\;\mathrm{size}=\frac{\mathrm{Total}\;\mathrm{number}\;\mathrm{of}\;\mathrm{individuals}\;\mathrm{counted}\;\mathrm{in}\;\mathrm{all}\;\mathrm{sampled}\;\mathrm{quardrats}}{\mathrm{Total}\;\mathrm{sampling}\;\mathrm{area}\;\left(m^{2}\right)}$$

The number of species per m^2^ is a measure of species richness, which refers to the count of different species present in each area. This metric is essential for understanding biodiversity within a specific habitat. By combining these metrics, a comprehensive understanding of both the current and future dynamics of an ecosystem can be determined.

### 2.4. Experimental Design and Statistical Analysis

The data on seedling emergence collected from each sample were pooled and converted to seeds per square meter for each quadrat to calculate the mean germinable seed densities for statistical analysis. The seedling datasets of riceflower were analysed using two-way analysis of variance (ANOVA). The experimental design employed a factorial design, comparing the effects of cultivated vs. pasture paddocks at upper and lower soil depths, years vs. sites, and interactions among these treatments. This statistical approach allowed for the assessment of how these factors influenced the mean germinable seed densities of riceflower, providing insights into the impact of different agricultural practices and soil depths on the soil seedbank. The results were presented as the total number of germinable seeds per square meter for all species, as it was impractical to cover each species individually. The summation of seed counts was intended as an aggregate measure to assess the overall seedbank density across the study period.

## 3. Results

**Figure 2:**
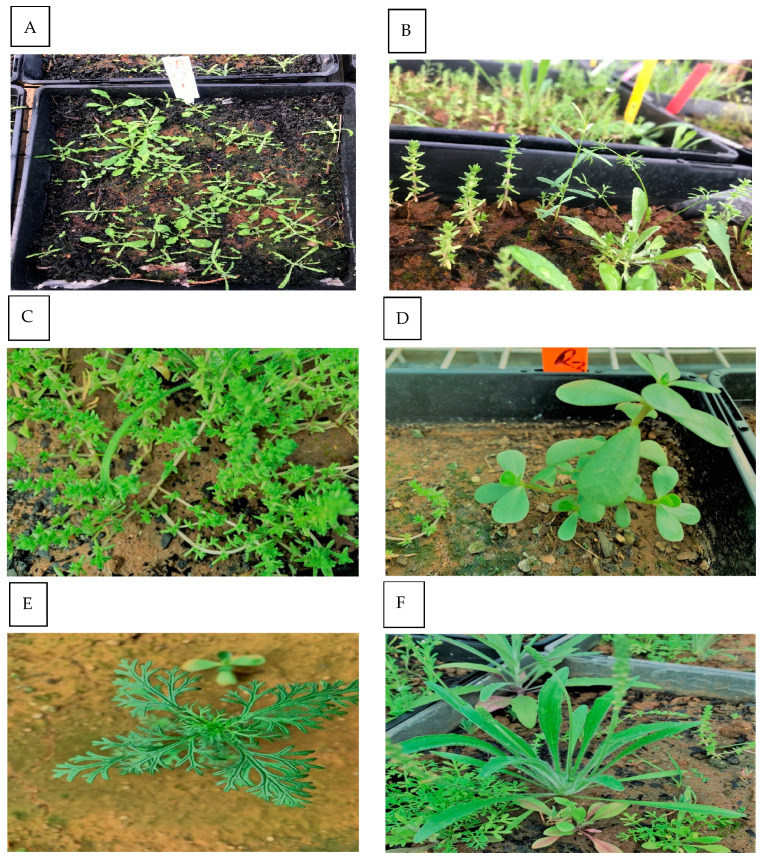
The most dominant species observed in the seedbank trial. Key plant species observed in the study: (**A**,**B**) Annual riceflower, (**C**) *Crassula sieberiana*, (**D**) *Portulaca oleracea*, (**E**) *Daucus glochidiatus*, and (**F**) *Plantago cunninghamii*.

Figure 2 highlights the distinct morphological features and growth patterns of the most dominant plant species observed in this study. Annual riceflower, represented in panels (A) and (B), is characterized by its slender stems and dense clusters of small white- to cream-coloured flowers. *Crassula sieberiana* (Schult. & Schult.f.) Druce (C) stands out with its fleshy, succulent leaves and clustered growth form, allowing it to thrive in arid and nutrient-poor environments. *Portulaca oleracea* L. (D), commonly known as purslane, is identifiable by its thick, paddle-shaped leaves and small yellow flowers, demonstrating resilience in disturbed and compacted soils. *Daucus glochidiatus* Labill. (E) is notable for its feathery leaves and umbrella-shaped inflorescence, which enable it to occupy less competitive, open environments. Finally, *Plantago cunninghamii* Decne. (F) is distinguished by its elongated, narrow leaves and low-growing rosette form, making it well adapted to grazing pressure and resource-limited conditions. Together, these species illustrate a range of growth pattern and adaptations to varying soil and environmental conditions, reflecting their ecological roles in pasture and cultivated paddocks.

### 3.1. The Seedbank Species Diversity at Site 1

There was a significant difference (*p* < 0.05) in the number of germinable annual riceflower seeds found between the pasture and cultivated paddock at the two seedbank depths and between the 2 years of study (Tables S1–S3).

At Site 1 in 2019, the highest germinable *Pimelea* seed density (134 m^−2^) was recorded in the topsoil (0–5 cm) of the pasture paddock, with *Crassula sieberiana* dominating (930 m^−2^), followed by *Plantago cunninghamii* (363 m^−2^) and *Oenothera indecora* (197 m^−2^) (Table 4). In the lower layer (5–10 cm), *Pimelea* seed density decreased (32 m^−2^), but *C. sieberiana* remained dominant (1427 m^−2^). In the cultivated paddock, *Pimelea* seeds were more evenly distributed across layers, with 57 m^−2^ in the top (0–7.5 cm) and 51 m^−2^ in the lower (7.5–15 cm) layer, and *C. sieberiana* remained the most abundant species (Table 4).

In 2020, the topsoil (0–5 cm) of the pasture paddock again had the highest germinable *Pimelea* seed density (146 m^−2^), with *C. sieberiana* (446 m^−2^), *P. cunninghamii* (280 m^−2^), and *Cyperus eragrostis* (184 m^−2^) as the dominant species (Table 4). Fewer seeds were recorded in the lower layer (25 m^−2^). In the cultivated paddock, seed distribution was balanced, with 102 m^−2^ in the topsoil (0–7.5 cm) and 70 m^−2^ in the lower layer (7.5–15 cm), with *C. sieberiana* remaining dominant in both layers.

### 3.2. The Seedbank Species Abundance and Evenness at Site 1

In 2019, the pasture paddock’s topsoil (0–5 cm) had a SWI of 1.46, with 1803 germinable seeds, an average of 200.2 seeds for nine species m^−2^. The lower layer (5–10 cm) showed higher diversity (SWI 1.63), with 2978 seeds, an average of 297.8 for 10 species per m^−2^ (Table 5). In 2020, the topsoil SWI increased significantly to 2.26, with 1750 seeds, an average of 134.8 for 13 species m^−2^, while the lower layer’s SWI dropped to 1.05, with 115 seeds, an average of 31.67 for three species m^−2^ (Table 5). In the cultivated paddock in 2019, the top layer (0–7.5 cm) had an SWI of 1.20, 872 seeds with an average of 97.0 for nine species m^−2^. The lower layer (7.5–15 cm) had a slightly higher SWI of 1.26, 338 seeds with an average of 67.6 for five species m^−2^ (Table 5). In 2020, the cultivated paddock showed an increase in diversity. The topsoil (0–7.5 cm) had an SWI of 1.84, with 1019 germinable seeds, averaging 113.3 for nine species m^−2^. The lower layer (7.5–15 cm) had an SWI of 1.17 with 1579 seeds, averaging 197.2 for eight species m^−2^ (Table 5). Species diversity and evenness varied by soil depth and management, with the pasture paddock showing greater shifts in composition and the cultivated paddock displaying more uniform diversity.

### 3.3. The Seedbank Species Diversity at Site 2

In 2019, the highest number of germinable annual riceflower seeds (108 m^−2^) was recorded in the topsoil (0–5 cm) of pasture paddocks at Site 2, with Australian stonecrop dominating (529 m^−2^) (Table 6). No riceflower seeds were found in the lower layer (5–10 cm). In cultivated paddocks, riceflower seeds were present in both layers, with more in the top layer (127 m^−2^) (Table 6). Australian stonecrop dominated the topsoil, while Australian carrot had the highest seed density in the lower layer. In 2020, annual riceflower seeds in pasture paddocks were again confined to the topsoil (121 m^−2^), with Australian stonecrop remaining dominant (634 m^−2^) (Table 6). In cultivated paddocks, riceflower seeds were present in both layers, with higher counts in the topsoil (140 m^−2^), while Australian stonecrop dominated the lower layer. Across 2019–2020, pasture paddocks had a total riceflower seed count of 229 m^−2^, while cultivated paddocks showed a greater increase with 401 m^−2^ (Table 6).

### 3.4. The Species Abundance and Evenness at Site 2

In 2019 at Site 2, the pasture paddock exhibited an SWI of 1.62 in the topsoil (0–5 cm) with 1038 germinable seeds m^−2^, with an average population size of 94.4 m^−2^ for 11 species. The lower soil layer (5–10 cm) had a lower SWI of 1.09, 522 germinable seeds m^−2^, with an average of 87 m^−2^ for six species. In 2020, the topsoil layer showed an increase in SWI to 1.82, with 1420 germinable seeds m^−2^, an average population size of 129.1 m^−2^ for 11 species (Table 7). The lower layer’s diversity slightly improved with an SWI of 1.13 and 898 germinable seeds m^−2^, averaging 149.7 m^−2^ for 6 species (Table 7).

In the cultivated paddock, the 2019 topsoil layer (0–7.5 cm) had an SWI of 1.52, 1817 germinable seeds m^−2^, with an average population of 227 per m^−2^ for eight species. The lower layer (7.5–15 cm) showed an SWI of 1.36, with 789 germinable seeds m^−2^, with an average population size of 157.8 m^−2^ for five species. In 2020, the topsoil layer’s SWI decreased to 1.30, despite an increase in germinable seeds (2758 per m^2^), with an average of 459.5 m^−2^ for six species (Table 7). The deeper layer’s SWI rose to 1.34, with 828 germinable seeds m^−2^, averaging 165.6 m^−2^ for five species (Table 7).

### 3.5. Comparison of Species Diversity, Abundance, and Evenness Between Sites and Years

Comparing species diversity and richness across the two sites and years reveals notable differences (Table 5 and Table 7). In 2019, the pasture paddock at Site 1 had lower diversity (1.46) in the upper soil layer compared to Site 2 (1.62). However, Site 1’s diversity significantly increased to 2.26 in 2020, indicating improved species evenness and richness. In contrast, Site 1’s lower soil layer saw a decrease from 1.63 in 2019 to 1.05 in 2020, while Site 2’s pasture showed more stable diversity, with slight increases in both layers (1.62 to 1.82 in the upper and 1.09 to 1.13 in the lower layer).

In the cultivated paddocks, Site 1 exhibited lower diversity in 2019 (1.20 and 1.26 for the upper and lower layers, respectively) compared to Site 2 (1.52 and 1.36). By 2020, Site 1’s upper soil layer diversity increased to 1.84, while the lower layer decreased slightly to 1.17. The diversity of Site 2 in the upper layer decreased from 1.52 in 2019 to 1.30 in 2020, with the lower layer remaining stable at 1.34.

## 4. Discussion

The total number of germinable riceflower seeds across all soil depths showed considerable variation between years and land uses. The main goal of this study was to explore the seedbank dynamics of annual riceflower, focusing on how soil conditions influence soil seedbanks. At both sites, the average soil pH was 6.01, accompanied by moderate levels of organic matter and essential nutrients, suggesting that these conditions are conducive to the persistence and germination of annual riceflower seeds. The strong correlation between soil organic carbon and total nitrogen is vital, as these elements play a key role in soil fertility and weed seedbank dynamics [17]. The higher carbon-to-nitrogen ratio observed in the cultivated paddocks at Site 2 points to slower decomposition rates and nitrogen release, which may affect plant growth and soil health differently compared to Site 1.

The pasture paddock at Site 1 showed fluctuations in the number of germinable annual riceflower seeds between 2019 and 2020, with an overall increase in the topsoil layer and a decrease in the lower soil layer. Australian stonecrop was the dominant species in both years and across both soil layers in the pasture paddock. In pasture paddocks, most annual riceflower seeds are concentrated in the topsoil layer (0–5 cm), consistent with previous research indicating that weed seeds remain in the uppermost layers due to dispersal mechanisms and surface disturbances [18,19] In contrast, cultivated paddocks show a more even distribution of seeds across both soil depths, suggesting that cultivation buries seeds deeper, potentially reducing their emergence.

Seedbank data from Site 1 indicate that annual riceflower is present in moderate to high numbers, primarily in the topsoil. Cultivation practices, which mechanically disturb the soil, likely stimulate germination or destroy seeds, thereby reducing the overall seedbank. This redistribution affects germination dynamics and emergence patterns, enhancing seed densities due to improved aeration and temperature fluctuations breaking seed dormancy [20,21]. Additionally, cultivation can increase seed densities and nutrient availability, supporting seedling growth and competitiveness [22]. The redistribution of seeds during cultivation plays a crucial role in shaping germination dynamics and emergence patterns by modifying soil conditions. As seeds are brought closer to the surface or shifted within the soil profile, they encounter improved aeration, increased exposure to light, and greater temperature fluctuations—key factors that help break dormancy and trigger germination. Temperature changes mimic natural seasonal cycles, signalling to seeds that conditions are suitable for growth. Additionally, cultivation loosens compacted soil, enhancing water infiltration and root development, which further supports seedling emergence. This process often results in higher seed densities in the upper soil layers, where germination is more likely to occur. By creating a more favourable environment for seeds, cultivation can increase overall seed emergence, benefiting both crops and weeds. This highlights the dual impact of soil disturbance, promoting productivity while also potentially encouraging the spread of competitive species.

While pasture paddocks maintain higher species diversity, cultivation results in a higher proportion of annual riceflower seeds at greater depths, which can aid in managing its emergence. Increased diversity in the topsoil of cultivated paddocks suggests that tillage can improve soil conditions and promote a diverse plant community [23]. However, topsoil diversity decline at other sites indicates potential dominance by fewer species, highlighting the trade-offs of different land management practices [24]. These findings suggest that land management practices, such as cultivation, can significantly influence the distribution and abundance of seeds. The higher seed counts observed in the cultivated paddock indicate a potential challenge for management that should be addressed through integrated strategies.

No-till fields have been shown to increase the number, diversity, or activity of seed-consuming fauna compared to conventionally tilled fields [25] and this could be attributed to several factors. One of the main reasons for this is the increased habitat availability provided by no-till fields, which offers more refuge for a wide variety of seed predators. These predators are essential for regulating seed populations and, as such, contribute to the reduction of weed seedbanks. No-till fields provide a more stable and diverse environment, supporting a broader range of organisms that consume seeds, such as insects, rodents, and birds [26]. This greater habitat diversity can lead to increased seed predation, which plays a critical role in controlling unwanted plant species and enhancing the sustainability of agricultural ecosystems. Additionally, no-till farming can lead to a decreased mortality rate of these seed-consuming faunas. Unlike conventional tillage systems, which disrupt the soil and destroy habitats, no-till fields offer a more protected environment. As a result, seed predators can persist longer, increasing their activity and effectiveness in controlling weed seed populations [27]. This effect is particularly notable in areas with a high density of weed species, where the predation of weed seeds becomes a significant factor in seedbank management.

Seeds with smaller sizes have been documented to generally become buried deeper in the soil, which helps them to avoid germination due to a state of quiescence. This occurs because smaller seeds typically lack a light requirement, which is a key trigger for seedling emergence [24,28]. However, this relationship between seed size and burial depth cannot be universally applied across all species and environments. Other factors such as soil texture, moisture, and organic matter play a crucial role in determining seed burial depth and germination patterns [29,30]. Soil characteristics, such as compaction and the presence of specific microbial communities, can influence seed burial and subsequent dormancy by either promoting or hindering seedling establishment [19,31]. Moreover, the longer survival of seeds in the soil cannot be attributed solely to their size. Other factors including seed coat properties, physiological dormancy mechanisms, and environmental conditions also contribute to the longevity of seeds in the soil seedbank [32]. Therefore, while seed size is an important factor influencing seed behaviour, it should be considered alongside a range of environmental and ecological factors that interact in complex ways.

Previous research has indicated that annual riceflower seed exhibits complex dormancy mechanisms, with germination and emergence influenced by multiple factors including optimal environmental conditions (light, temperature, and rainfall) and burial depths. These factors contribute to asynchronous germination events, as documented by [6]. This complexity in seed dormancy and germination behaviour highlights the challenges in managing riceflower, as predicting and controlling its emergence requires a comprehensive understanding of its ecological requirements and responses to varying environmental conditions [6]. However, as the seedbank trial extended over 12 weeks, it is reasonable to assume that most seeds encountered favourable conditions for germination at some point during the trial period.

Pasture paddocks generally exhibited higher species diversity and evenness, indicated by higher SWI values, compared to cultivated paddocks. Cultivated paddocks, although showing reduced diversity, had a higher proportion of annual riceflower seeds at greater depths, potentially reducing their emergence. This suggests that pasture paddocks maintain higher species richness and diversity, while cultivated paddocks support higher average population sizes at certain depths and years. Understanding these dynamics is essential for sustainable land management strategies that promote biodiversity and ecosystem resilience.

Cultivation impacts species diversity, with increased topsoil diversity in cultivated paddocks at Site 1 suggesting that tillage enhances soil conditions, promoting a diverse plant community. However, a decline in topsoil diversity at Site 2, despite increased seed densities, indicates potential species dominance [19]. Variability in seedbank dynamics (i.e., seed density and distribution) between 2019 and 2020, likely due to climatic differences, further underscores the importance of considering annual climatic variations in riceflower management strategies. At Site 1, the SWI in pasture paddocks increased from 1.46 in 2019 to 2.26 in 2020, indicating higher species evenness and richness in the second year, while the lower soil layer showed a decreased SWI, reflecting reduced species richness. Over time, the germinable seeds of annual riceflower in the pasture paddock increased slightly, whereas the cultivated paddock saw a substantial rise, suggesting that cultivation enhances the germinable seedbank through soil disturbance and seed redistribution [21]. These factors collectively create a microenvironment that promotes seedling growth and increases the density of the seedbank in cultivated areas, whereas pasture paddocks, with minimal disturbance, see slower and less pronounced seedbank growth. These trends suggest that Site 1 experienced more dynamic changes in species diversity, particularly in response to different land management practices, whereas Site 2 maintained more consistent diversity levels, with some declines in the cultivated upper soil layer. Both sites showed similar seedbank patterns, with notable differences in species diversity and richness due to land use and annual climatic variations. Cultivation influenced seed distribution, reducing diversity but potentially managing annual riceflower by burying seeds deeper.

The comparison between pasture and cultivated paddocks highlights the differing impacts of these land management practices on seedbanks, soil health, and plant diversity. Pasture paddocks, characterized by minimal soil disturbance and continuous vegetation cover, create conditions that promote biodiversity and contribute to a diverse seedbank. The stable soil structure supports natural plant growth, enhances microbial activity, and reduces erosion. However, over time, this undisturbed environment may allow weed species such as annual riceflower to gradually increase and dominate, if not properly managed. In contrast, cultivated paddocks experience soil disturbance, which redistributes seeds and can suppress surface-germinating species by burying their seeds too deeply for germination. While cultivation can help manage aggressive species, it often reduces overall plant diversity by favouring species that thrive under disturbed conditions. Additionally, continuous cultivation can lead to soil degradation, erosion, and nutrient loss. Therefore, balancing pasture and cultivation practices is essential to maintaining soil health, managing invasive species, and promoting long-term agricultural productivity.

## 5. Conclusions

This study underscores the importance of understanding seed distribution and species diversity in managing *Pimelea*, particularly in pasture and cultivated paddocks. Farmers can use these findings to refine management strategies aimed at controlling *Pimelea* infestations. In pasture paddocks, where higher species diversity and evenness are observed, reducing soil disturbance can help maintain this diversity, potentially suppressing the growth of *Pimelea*. Conversely, in cultivated paddocks, where *Pimelea* seeds tend to be redistributed deeper in the soil, farmers may need to adjust cultivation practices, such as minimizing soil disturbance, to prevent the deeper burial of seeds that could lead to future infestations. Additionally, the year-to-year variability in seedbank dynamics highlights the need for farmers to consider environmental factors in their management strategies. A combination of rotational grazing, targeted herbicide application, and strategic cultivation practices, alongside monitoring climatic conditions, will provide a more comprehensive approach to *Pimelea* management. Future research could further guide farmers on specific practices to reduce *Pimelea* seedbanks while fostering long-term biodiversity and resilience in their farming systems.

## Figures and Tables

**Table 1:** Soil physio-chemical properties of annual riceflower-invaded paddocks (taken in the range of 0 to 10 cm depth) at two sites and from two land use areas.

Site	Land Use	Coordinates		pH	Organic Matter (%)	K (mg kg^−1^)	P (mg kg^−1^)	S (mg kg^−1^)	N (%)	C (%)	C/N Ratio
Site 1	Pasture	27°38′60″ S	148°42′29″ E	6.22	2.00	443	14.0	3.0	0.095	1.38	14.53
	Cultivated	27°38′21″ S	148°42′43″ E	5.69	1.31	396	21.4	3.7	0.073	0.84	11.51
Site 2	Pasture	27°15′32″ S	148°34′26″ E	6.05	1.65	265	13.9	2.8	0.094	1.09	11.60
	Cultivated	27°13′90″ S	148°32′58″ E	6.08	2.26	380	43.8	1.0	0.116	1.98	17.07
Mean				6.01	1.80	371	23.2	2.6	0.094	1.32	14.04

Potassium (K), Sulphur (S), Nitrogen (N), Phosphorus (P), and Carbon (C).

**Table 2:** Monthly rainfall data recorded at the two study sites over the 2 years of study and for the year previous to the study. Shaded months indicate peaks in rainfall for that year.

Sites	Year	Rainfall (mm)												Total
		Jan	Feb	Mar	Apr	May	Jun	Jul	Aug	Sep	Oct	Nov	Dec	
Site 1	2018	1	68	113	4	0	12	2	34	2	81	56	34	408
	2019	0	0	32	47	15	4	11	8	0	5	26	4	153
	2020	41	211	52	5	20	0	8	21	13	33	1	17	422
Site 2	2018	0	57	53	4	0	13	17	33	18	58	47	37	337
	2019	0	4	68	53	0	6	4	3	4	0	10	0	152
	2020	80	343	30	7	20	9	9	23	25	51	0	49	646

**Table 3:** Average monthly temperature recorded at the two study sites over the 2 years of study. Shaded months indicate the highs and lows in average day and night temperatures for that year.

Sites	Year	Temperature (°C)												Annual Mean
		Jan	Feb	Mar	Apr	May	Jun	Jul	Aug	Sep	Oct	Nov	Dec	
Site 1	Max	35.3	33.8	32.0	28.0	23.3	19.9	19.7	22.2	26.5	29.8	32.2	34.2	28.0
	Min	22.4	21.2	19.0	13.9	9.0	6.4	5.3	6.2	10.7	14.7	18.1	20.5	13.9
Site 2	Max	34.4	33.6	31.8	28.4	23.8	20.4	19.9	22.0	26.0	29.4	31.9	33.7	27.9
	Min	21.0	20.4	18.1	13.6	8.9	5.7	4.3	5.6	9.5	14.0	17.2	19.5	13.1

**Table 4:** The number of germinable seeds of each species present m^−^^2^, taken from two soil depths and from two land uses (pasture and cultivated) at Site 1 in 2019 and 2020.

Land Type	Soil Depth (cm)	Species	Family	Growth Form	Germinable Seeds (m^−2^)	
					2019	2020
Pasture paddock	0.0 to 5.0	*Asphodelus tenuifolius* Cav.	Asphodelaceae	P *	13	0
		*Conyza canadensis* L.	Asteraceae	A	0	45
		*Crassula sieberiana* (Schult. & Schult.f.) Druce	Crassulaceae	AS	930	446
		*Cyperus eragrostis* Lam.	Cyperaceae	P	0	184
		*Daucus glochidiatus* Labill.	Apiaceae	AF	70	96
		*Hypochaeris microcephela* Sch.Bip.	Asteraceae	A	0	64
		*Linaria purpurea* (L.) Mill.	Plantaginaceae	P	0	95
		*Oenothera indecora* W.Dietr.	Onagraceae	A/B	197	140
		*Oenothera laciniata* Hill.	Onagraceae	A/B	0	70
		*Oxalis corniculata* L.	Oxalidaceae	PF	76	115
		*Pimelea trichostachya* Lindl.	Thymelaeaceae	A	134	146
		*Plantago cunninghamii* Decne.	Plantaginaceae	AH	363	280
		*Portulaca oleracea* L.	Portulacaceae	AS	13	64
		*Soliva anthemifolia* Juss.	Asteraceae	AF	6	6
		Total			1803	1750
	5.0 to 10.0	*Apium leptophyllum* Pers.	Apiaceae	A	229	0
		*Crassula sieberiana* (Schult. & Schult.f.) Druce	Crassulaceae	AS	1427	45
		*Daucus glochidiatus* Labill.	Apiaceae	AF	153	0
		*Hypochaeris microcephela* Sch.Bip.	Asteraceae	A	38	0
		*Oenothera indecora* W.Dietr.	Onagraceae	A/B	306	0
		*Oxalis corniculata* L.	Oxalidaceae	PF	185	45
		*Pimelea trichostachya* Lindl.	Thymelaeaceae	A	32	25
		*Plantago cunninghamii* Decne.	Plantaginaceae	AH	522	0
		*Portulaca oleracea* L.	Portulacaceae	AS	45	0
		*Soliva anthemifolia* Juss.	Asteraceae	AF	38	0
		Total			2978	115
Cultivated paddock	0.0 to 7.5	*Apium leptophyllum* Pers.	Apiaceae	A	0	96
		*Asphodelus tenuifolius* Cav.	Asphodelaceae	P	0	102
		*Conyza canadensis* L.	Asteraceae	A	32	0
		*Crassula sieberiana* (Schult. & Schult.f.) Druce	Crassulaceae	AS	599	433
		*Daucus glochidiatus* Labill.	Apiaceae	AF	19	51
		*Hypochaeris microcephela* Sch.Bip.	Asteraceae	A	13	89
		*Oenothera indecora* W.Dietr.	Onagraceae	A/B	13	0
		*Oxalis corniculata* L.	Oxalidaceae	PF	51	0
		*Pimelea trichostachya* Lindl.	Thymelaeaceae	A	57	102
		*Plantago cunninghamii* Decne.	Plantaginaceae	AH	0	32
		*Portulaca oleracea* L.	Portulacaceae	AS	76	64
		*Soliva anthemifolia* Juss.	Asteraceae	AF	13	51
		Total			872	1019
	7.5 to 15.0	*Campanula patula* L.	Campanulaceae	B	0	82
		*Conyza canadensis* L.	Asteraceae	A	0	32
		*Crassula sieberiana* (Schult. & Schult.f.) Druce	Crassulaceae	AS	172	1089
		*Daucus glochidiatus* Labill.	Apiaceae	AF	83	0
		*Dysphania schraderiana* (Schult)	Amaranthaceae	A	0	38
		*Hypochaeris microcephela* Sch.Bip.	Asteraceae	A	0	51
		*Oxalis corniculata* L.	Oxalidaceae	PF	0	159
		*Pimelea trichostachya* Lindl.	Thymelaeaceae	A	51	70
		*Plantago cunninghamii* Decne.	Plantaginaceae	AH	19	0
		*Portulaca oleracea* L.	Portulacaceae	AS	0	57
		*Soliva anthemifolia* Juss.	Asteraceae	AF	13	0
		Total			338	1579

* P = Perennial, A = Annual, B = Biennial, AS = Annual shrub, AH = Annual herb, AF = Annual forb, and PF = Perennial forb.

**Table 5:** Shannon–Weiner Index of species richness, the number of germinable seeds, the average population size of the germinable soil seedbank, and the number of species present. The data were collected from soil samples taken to a depth of 10 cm in pasture and to 15 cm in cultivated paddocks at Site 1 during 2019 and 2020.

Year	Land Type	Soil Depth (cm)	Number of Germinable Seeds (m^−2^)	Average Population Size (m^−2^)	Number of Species Present (m^−2^)	Shannon–Weiner Index
2019	Pasture paddock	0.0 to 5.0	1803	200.2	9	1.46
		5.0 to 10.0	2978	297.8	10	1.63
	Cultivated paddock	0.0 to 7.5	872	97.0	9	1.20
		7.5 to 15.0	338	67.6	5	1.26
2020	Pasture paddock	0.0 to 5.0	1750	134.8	13	2.26
		5.0 to 10.0	115	31.7	3	1.05
	Cultivated paddock	0.0 to 7.5	1019	113.3	9	1.84
		7.5 to 15.0	1579	197.2	8	1.17

**Table 6:** The number of germinable seeds of each species present m^−^^2^, taken from two soil depths and from two land uses (pasture and cultivated) at Site 2 during 2019 and 2020.

Land Type	Depth (cm)	Species	Family	Growth Form	Germinable Seeds (m^−2^)	
					2019	2020
Pasture paddock	0.0 to 5.0	*Apium leptophyllum* F.Muell.	Apiaceae	A *	38	45
		*Asphodelus tenuifolius* Cav.	Asphodelaceae	P	25	51
		*Cardamine dentata* Schult.	Brassicaceae	A	13	25
		*Crassula sieberiana* (Schult. & Schult.f.) Druce	Crassulaceae	AS	529	637
		*Daucus glochidiatus* Labill.	Apiaceae	AF	38	96
		*Erodium crinitum* L.	Geraniaceae	A/B	0	13
		*Fumaria indica* L.	Papaveraceae	H	19	0
		*Gamochaeta americana* Mill.	Asteraceae	A	32	57
		*Oxalis corniculata* L.	Oxalidaceae	PF	32	159
		*Pimelea trichostachya* Lindl.	Thymelaeaceae	A	108	121
		*Portulaca oleracea* L.	Portulacaceae	AS	191	178
		*Soliva anthemifolia* Juss.	Asteraceae	AF	13	38
		Total			1038	1420
	5.0 to 10.0	*Calotis squamigera* C.T.White	Asteraceae	AH	19	19
		*Crassula sieberiana* (Schult. & Schult.f.) Druce	Crassulaceae	AS	293	503
		*Erodium crinitum* L.	Geraniaceae	A/B	6	19
		*Oxalis corniculata* L.	Oxalidaceae	PF	13	45
		*Phyllanthus amarus* Schum. & Thonn.	Phyllanthaceae	A	25	38
		*Portulaca oleracea* L.	Portulacaceae	AS	166	274
		Total			522	898
Cultivated paddock	0.0 to 7.5	*Avena sativa* L.	Poaceae	A	19	0
		*Crassula sieberiana* (Schult. & Schult.f.) Druce	Crassulaceae	AS	306	1134
		*Daucus glochidiatus* Labill.	Apiaceae	AF	904	764
		*Gamochaeta americana* Mill.	Asteraceae	A	38	0
		*Oxalis corniculata* L.	Oxalidaceae	PF	64	140
		*Pimelea trichostachya* Lindl.	Thymelaeaceae	A	127	38
		*Plantago cunninghamii*	Plantaginaceae	AH	243	19
		*Portulaca oleracea* L.	Portulacaceae	AS	115	662
		Total			1817	2758
	**7.5 to 15.0**	*Amaranthus viridis* L.	Amaranthaceae	AH	6	25
		*Daucus glochidiatus* Labill.	Apiaceae	AF	503	382
		*Oxalis corniculata* L.	Oxalidaceae	PF	51	102
		*Pimelea trichostachya* Lindl.	Thymelaeaceae	A	19	115
		*Portulaca oleracea* L.	Portulacaceae	AS	210	204
		Total			790	828

* P = Perennial, A = Annual, B = Biennial, AS = Annual shrub, AH = Annual herb, AF = Annual forb, and PF = Perennial forb.

**Table 7:** Shannon Weiner Index of species richness, the number of germinable seeds, the average population size of the germinable soil seedbank, and the number of species present. The data were recorded from soil samples taken to a depth of 10 cm in pasture and to 15 cm in cultivated paddocks at Site 2 during 2019 and 2020.

Year	Land Type	Depth (cm)	Number of Germinable Seeds (m^−2^)	Average Population Size (m^−2^)	Number of Species (m^−2^)	Shannon–Weiner Index
2019	Pasture paddock	0.0 to 5.0	1038	94.4	11	1.62
		5.0 to 10.0	522	87	6	1.09
	Cultivated paddock	0.0 to 7.5	1817	227	8	1.52
		7.5 to 15.0	790	157.8	5	1.36
2020	Pasture paddock	0.0 to 5.0	1420	129.1	11	1.82
		5.0 to 10.0	898	149.7	6	1.13
	Cultivated paddock	0.0 to 7.5	2758	459.5	6	1.30
		7.5 to 15.0	828	165.6	5	1.34

## Data Availability

Data are contained within the article and supplementary materials.

## Supplementary Materials

The following supporting information can be downloaded at: https://www.mdpi.com/article/10.3390/biology14020109/s1, Table S1: Effect of pasture and cultivation on the emergence of *P. trichostachya* in upper and lower layer at site 1; Table S2: Effect of pasture and cultivation on the emergence of *P. trichostachya* in upper and lower layer at site 2; Table S3: Effect of years and sites on the emergence of *P. trichostachya*.
