# Supplementary material for: Soil Seedbank Dynamics and Species Diversity in Pimelea-Infested Paddocks Under Pasture and Cultivated Conditions
Source: Biology (Basel). 2025 Jan 21;14(2):109. doi: 10.3390/biology14020109 (PMC11851602; doi:10.3390/biology14020109)
Supplement: Supplementary file 1 [file biology-14-00109-s001.zip › biology-3344665-supplementary.pdf]

Table S1. Effect of pasture and cultivation on emergence of P. trichostachya in upper and lower layer at site 1

Source	DF	SS	MS	F	P
Land types	1	24.8489	24.8489	17.3485	
Depths	1	852. 4687	852. 4687	7.5235	0.0001
Interaction	1	369.6905	369.6905		0.0076
Error	76	3758.7732	49.4575		
Error (adjusted)	77	3783.6221	49.1379		
Total	79	5005.7812			

Table S2. Effect of pasture and cultivation on emergence of P. trichostachya in upper and lower layer at site 2

Source	DF	SS	MS	F	P
Land types	1	292.1011	292.1011	Inf	
Depths	1	1825.6319	1825.6319	Inf	0.000
Interaction	1	164.3069	164.3069	Inf	0.000
Error	76	4531.6240	59.6266		
Error (adjusted)	77	0.0000	0.0000		
Total	79	6813.6628			

Table S3. Years and sites on the emergence of riceflower

Source	DF	SS	MS	F	P
Years	1	276.1268	276.1268	3.8735	0.0536
Sites	1	6.3390	6.3390		
Interaction	1	91.5352	91.5352	0.2645	0.3645
Error	156	11451.7830	73.4089		
Error (adjusted)	157	11458.1220	72.9817		
Total	159	11825.7840			
